# Recent advances in understanding breast cancer and emerging therapies with a focus on luminal and triple-negative breast cancer

**DOI:** 10.12688/f1000research.17542.1

**Published:** 2019-04-30

**Authors:** Georges El Hachem, Andrea Gombos, Ahmad Awada

**Affiliations:** 1 Department of Hematology and Medical Oncology, Saint George Hospital University Medical Center, University of Balamand, Beirut, Lebanon; 2 Oncology Medicine Department, Institut Jules Bordet, Université Libre de Bruxelles, Brussels, Belgium

**Keywords:** Luminal breast cancer, triple negative, heterogeneity, personalized treatment

## Abstract

Breast cancer is a global health issue. For decades, breast cancer was classified into many histological subtypes on the basis of microscopic and immunohistochemical evaluation. The discovery of many key genomic driver events involved in breast cancer carcinogenesis resulted in a better understanding of the tumor biology, the disease heterogeneity and the prognosis leading to the discovery of new modalities of targeted therapies and opening horizons toward a more personalized medicine. In recent years, many therapeutic options emerged in the field of metastatic breast carcinoma, especially for the luminal subtypes. They were able to transform the course of the disease while maintaining quality of life. However, the options are still limited for triple-negative breast cancer, but the better knowledge of its complex biology and the discovery of molecular targets are promising for more efficient novel therapies.

## Introduction

Breast cancer (BC) is the most common malignancy and the second leading cause of death in women worldwide ^1, 2^. It is known for its heterogeneity with different molecular and prognostic profiles, which create many therapeutic challenges ^3, 4^. For many decades, BC classification has depended on the cell morphology and immunohistochemical evaluation of hormone receptors (HRs) and human epidermal growth factor 2 (HER2). Many other molecular biomarkers were assessed as predictive or prognostic factors. During the European Society of Medical Oncology (ESMO) 2018 congress, many practice-changing results related to the management of breast cancer were presented. The four major pre-existing molecular subtypes of BC are being remodeled into the following subgroups (in the same perspective of the St. Gallen International Expert Consensus Conference classification): luminal (HR ^+^) subtype classified according to the phosphoinositide 3-kinase (PI3K) status: wild-type or mutated; HER2 ^+^ disease; triple-negative subtype (HR ^−^/HER2 ^−^) currently subclassified according to the programmed death ligand 1 (PDL-1) status, either ≥1 (positive on immune cells) or 0 (negative) with the creation of quadruple negative entity when PDL-1 is absent; and breast cancer gene ( *BRCA*) mutated cancers ^5– 7^. In the era of precision medicine, recent advances in molecular profiling and genomic sequencing improved the understanding of BC and led to emerging therapies ^8^. The purpose of this short review is to answer how the understanding of metastatic BC (mBC) biology and heterogeneity over the last year resulted in the emergence of new targets and therapeutic options mainly in metastatic luminal and triple-negative breast cancers (TNBCs). This article will cover the different drugs that were investigated in phase III trials and will briefly mention other future perspectives. (Neo)adjuvant approaches and the therapeutic approach of HER2 disease will not be covered in this article since there was recently no significant therapeutic breakthrough.

## Advances in understanding breast cancer biology

BC is a heterogenic disease with a high potential of biological alterations through the course of the disease. Alike other cancer types, the recent advances in tumor sequencing technologies resulted in the identification of molecular targets and pathways involved in the carcinogenesis process of BC and disease progression ^9, 10^. Moreover, biomarkers were identified, predicting drug sensitivity or resistance: HR expression, HER2 amplification or mutation, mutations in estrogen receptor gene (ESR1), PI3K, and fibroblast growth factor receptor (FGFR) copy number alterations ^11^.

However, despite the presence of these biomarkers on tumor cells, resistance to corresponding developed targeted therapies can occur. One possible explanation consists of the high inter- and intra-tumoral heterogeneities. Intra-tumor heterogeneity has been evaluated because of the major advances in molecular biology and sequencing techniques. It is caused by a continuous spatial and temporal evolution. Besides genetics, tumor heterogeneity can result from epigenetic regulation or interaction with the tumor microenvironment ^10– 13^. Single tumor biopsy samples might miss rare spatially or temporally separated subclones, affecting treatment outcome. To address this issue, researchers started testing liquid biopsies (as circulating tumor cells or circulating free DNA) as a tool to understand the disease heterogeneity and the changes occurring upon progression. Despite some current limitations in early and metastatic settings, they are promising as reliable methods for detecting driver and targetable mutations or alterations and dynamic monitoring of treatment response and resistance ^14– 18^.

## Emerging therapies in breast cancer

### Luminal (HR ^+^) breast cancer

HR ^+^ BC is the most common subtype (around 60% of the cases). Endocrine therapy (ET) is the mainstay of treatment of this type of BC in adjuvant and metastatic settings. ET alone can be an effective option even in the presence of visceral metastases unless there is an extensive symptomatic visceral involvement or proof of endocrine resistance ^19^. ET consists of either depleting the estrogen (via oophorectomy, luteinizing hormone-releasing hormone [LHRH] agonists, or aromatase inhibitors [AIs]) or targeting the estrogen receptor (ER) (with the use of selective estrogen receptor modulators [SERMs] or more recently studied selective estrogen receptor down-regulators [SERDs]). In the last decade, several attempts have combined the known ETs with new targeted therapies in order to tackle or delay the resistance to hormonal therapy. The two main areas of research in this setting are the inhibition of the mammalian target of rapamycin/PIK3CA (mTOR/PIK3CA) pathway by specific inhibitors (everolimus and alpelisib) and intervening in the cell cycle progression by targeting cyclin-dependent kinase 4/6 (CDK4/6). Three CDK4/6 inhibitors have been approved in metastatic HR ^+^/HER2 ^−^ BC: palbociclib, ribociclib, and abemaciclib as first-line treatments in association with AIs (PALOMA-2, MONALEESA-2, and MONARCH-3) or as second-line therapies associated with fulvestrant (PALOMA-3, MONALEESA-3, and MONARCH-2). One trial addressed only pre-menopausal patients who received goserelin, and AI or tamoxifen combined with ribociclib obtained the same magnitude of benefit (MONALEESA-7). Through the pivotal trials, it has been shown that the early introduction of these targeted treatments with AIs resulted in a progression-free survival (PFS) gain of about 10 months, a consistent significant hazard ratio ranging between 0.55 and 0.57, and an improvement in overall response rate (ORR) ^20– 23^. Health-related quality of life is maintained over all of the first-line trials ^24, 25^ and the side effects consisting mainly of hematological toxicity which are easily manageable, thus favoring the first-line combination therapy. Moreover, CDK4/6 inhibitors showed positive results beyond first-line settings when associated with fulvestrant: there was a consistent PFS gain of about 5 to 7 months and a consistent significant hazard ratio ranging from 0.50 to 0.55 ^26– 29^. Moreover, the first data concerning the overall survival (OS) were reported in the PALOMA-3 trial, which showed a significant improvement in the median OS from 28 months with fulvestrant plus placebo to 34.9 months with fulvestrant plus palbociclib (hazard ratio 0.79; *P* = 0.0246) ^30^. Nevertheless, there are some differences in the safety profile among the three CDK4/6 inhibitors: abemaciclib was associated with less grade 3 or 4 neutropenia (21% in the MONARCH-3 trial compared with 66% and 59% in the PALOMA-2 and MONALEESA-2 trials, respectively), more grade 3 or 4 diarrhea (9.5% in the MONARCH-3 trial compared with 1.4% and 1.2% in the PALOMA-2 and MONALEESA-2 trials, respectively) and with thromboembolic events (4% of patients) ^22, 31, 32^. With ribociclib, a risk of QTc prolongation and liver toxicity has been reported ^19^. Interestingly, abemaciclib showed promising single-agent activity, and possibly an activity against brain metastases knowing its ability to cross the blood–brain barrier ^33, 34^. Unfortunately, there have been no clinical predictive biomarkers for response to CDK4/6 inhibitors ^26, 35^.

**Figure 1.**
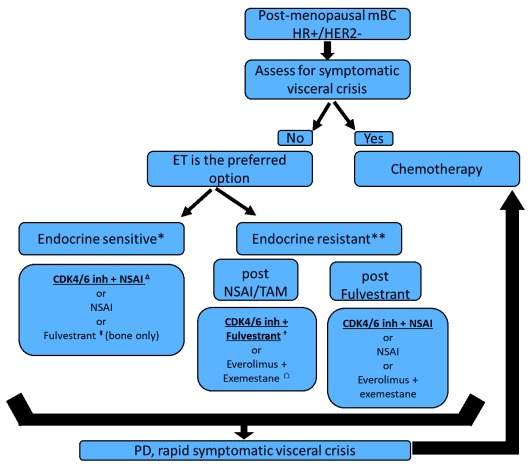
Current endocrine therapy in case of post-menopausal metastatic luminal breast cancer according to several pivotal trials. ∆, PALOMA-2, MONARCH-3, MONALEESA-2 trials; †, PALOMA-3, MONARCH-2, MONALEESA-3 trials; ‡, FALCON; ⌂, BOLERO-2 trial. The options printed in bold and underlined are the preferred options. For pre-menopausal women, the same algorithm may apply, with adjunction of ovarian suppression or ablation. *Endocrine-sensitive metastatic breast cancer (mBC) is defined in this algorithm as *de novo* luminal breast cancer or a disease that recurred more than 1 year after the end of adjuvant ET. **Endocrine-resistant mBC is defined as an mBC progressing while on ET or recurring less than 12 months after the end of adjuvant ET or during ET for metastatic disease. CDK4/6 inh, cyclin-dependent kinase 4/6 inhibitor; HER2 ^−^, human epidermal growth factor receptor 2–negative; HR ^+^, hormone receptor–positive; NSAI, non-steroidal aromatase inhibitor; PD, progressive disease; TAM, tamoxifen.

Another potential mechanism of resistance to ET is the activation of the PI3K-AKT-mTOR pathway conducting to cell survival. In the pivotal BOLERO-2 phase III trial, it was demonstrated that everolimus (mTOR inhibitor) with exemestane prolonged the PFS and increased the ORR as compared with exemestane alone after progression on AIs: gain in PFS of about 4 months with a hazard ratio of 0.43 (95% confidence interval [CI] 0.35–0.54; *P* <0.001) ^36^. Figure 1 proposes the current standard of care in metastatic luminal BC. This proposed algorithm is currently challenged by the positive results of SOLAR-1 trial alpelisib, an alpha-selective PI3K pathway inhibitor, in combination with fulvestrant in PI3K-mutated luminal mBC (40% of the luminal population have PI3K mutation) ^37, 38^.

However, many challenges remain in providing treatment for this population. Most of the trials did not include pre-menopausal women, but most of the consensuses recommend the same treatment as for post-menopausal women with ovarian suppression or ablation. Despite this impressive benefit seen with CDK4/6 inhibitors, resistance can still occur. The correct sequencing of ET and targeted treatment association is still an unanswered issue because the mTOR inhibitor trials did not include patients pre-treated with CDK4/6 inhibitors and vice versa. Will the response to mTOR inhibitors be the same as it was before the era of CDK4/6 inhibitors? Addressing this issue in a prospective clinical trial will be challenging; thus, data collection and analysis in large existing phase III trials on the efficacy of treatments post-CDK4/6 inhibitors would be of utmost importance. The financial burden of these treatments should be addressed as well. Interestingly, many ongoing trials are evaluating the continuation of CDK4/6 inhibition beyond progression in advanced ER ^+^, HER2 ^−^ BC: MAINTAIN (NCT02632045), NCT02871791, TRINITI-1 (NCT02732119), PACE (NCT03147287), and NCT01857193. Also, several ongoing trials are testing another hypothesis: the combination of CDK4/6 inhibitors with different PI3K/mTOR inhibitors (NCT03128619, NCT03006172, NCT02684032, NCT02389842, NCT02732119, NCT02871791, and NCT02599714).

Among several alterations with potential clinical relevance, PIK3CA inhibitors combined with fulvestrant showed promising results at the expense of high toxicity profile. However, the newer selective PIK3CA inhibitors taselisib and alpelisib were tested in two randomized phase III trials—SANDPIPER and SOLAR-1, respectively—and met their primary endpoint with improvement in PFS and manageable toxicity profile. Table 1 summarizes the data from five trials with PIK3CA inhibitors.

**Table 1.** Different trials testing the PIK3CA inhibitors in post-menopausal metastatic luminal breast cancer.

Trial	Population: mBC HR ^+^ HER2 ^−^	Endocrine therapy	Number of patients	Results
BELLE-2 (phase III) ^51^	PD after AI (one line of chemotherapy in metastatic disease was allowed; design similar to that of PALOMA-3 trial)	Buparlisib + FVL versus FVL	1147	- Better mPFS in both PIK3CA mutated or wild-type • wild-type: mPFS increased from 4.5 to 6.8 months (hazard ratio 0.8; *P* = 0.0033) • mutated: mPFS increased from 4 to 6.8 months (hazard ratio 0.76; *P* = 0.0014) - Bad toxicity profile with 23% SAE in buparlisib group
BELLE-3 (phase III) ^52^	PD after mammalian target of rapamycin (mTOR) inhibitor	Buparlisib + FVL versus FVL	432	- mPFS increased from 1.8 to 3.9 months (hazard ratio 0.67; *P* = 0.00030) - Significant toxicity profile with 22% SAE in buparlisib group
FERGI (part 2 of phase II) ^53^	PD after AI (part 2 cohort including PI3KCA mutated tumors only)	Pictilisib + FVL versus FVL	61	- No statistically significant difference in mPFS - Significant toxicity profile with 36% of at least grade 3 AE and 5% SAE in pictilisib group
SANDPIPER (phase III) ^54^	PD after AI (PIK3CA-mutated tumors only)	Taselisib (selective PI3K inhibitor) + FVL versus FVL	516	- mPFS increased from 5.7 to 7.4 months (hazard ratio 0.7; *P* = 0.0037) - Taselisib group: at least grade 3 AEs: 12% diarrhea, 10% hyperglycemia, 3% colitis, 2% stomatitis, and treatment discontinuation in 17%
SOLAR-1 (phase III) ^37, 38^	PD after AI with or without a cyclin-dependent kinase 4/6 (CDK4/6) inhibitor	Alpelisib (α-specific PI3K inhibitor) + FVL versus FVL	572	- mPFS increased from 5.7 to 11 months (hazard ratio 0.65; *P* = 0.00065) in mutated tumors - Alpelisib group: grade 3 AE: 32.7% hyperglycemia, 9.9% rash, and 6.7% diarrhea

AE, adverse event; AI, aromatase inhibitor; FVL, fulvestrant; HER2 ^−^, human epidermal growth factor receptor 2–negative; HR ^+^, hormone receptor–positive; mBC, metastatic breast cancer; mPFS, median progression-free survival; PD, progression disease; SAE, serious adverse event.

ESR1 mutations can develop during disease evolution. This has been described mainly during treatment with AI and did not influence the effectiveness of mTOR and CDK4/6 inhibitors. In order to target ESR1 mutations, many SERDs are under evaluation: G1T48 (phase I; NCT03455270), RAD 1901 (phase IB; NCT02650817), AZD9496 (phase I; NCT03236974), GDC-0810 (phase II; NCT02569801), and SAR439859 (phase I; NCT03284957).

### Triple-negative breast cancer

TNBC is a subtype of breast carcinoma lacking the expression of HR and HER2. It accounts for 15 to 20% of BC and is known to be the most aggressive subgroup with a high risk of recurrence ^39, 40^. Metastatic TNBC is highly heterogenic and, despite all the advances in the field of BC, remains an unmet medical need where few therapies besides the standard cytotoxic chemotherapy are available ^41^. While sharing immunohistochemical characteristics, diverse molecular subtypes of TNBC have different gene expression profiles, clinical behavior, and response to treatment ^42^. Lehmann *et al*. ^41^ highlight the intrinsic diversity of TNBC by using gene expression profiling. They subclassify TNBC into five categories, each exhibiting a different treatment sensitivity: (1) basal-like 1, which shows a higher sensitivity to platinum-based chemotherapy and DNA damage therapies; (2) basal-like 2, which is less sensitive to chemotherapy and is characterized by an upregulation of genes involved in the growth factor signaling pathway; (3) the immune group; (4) mesenchymal with a great response to PI3K pathway inhibitors; and (5) luminal androgen receptor (LAR), which may be more sensitive to androgen receptor (AR) antagonists and have a relative insensitivity to standard chemotherapy ^42– 47^.

In this perspective of understanding the TNBC biology, the entity of *BRCA*-ness was elaborated to describe the tumors that are *BRCA*-proficient but act as if they are deficient in DNA double-strand break repair by homologous recombination. Certain similar defects in homologous recombination can be encountered after the methylation of *BRCA* gene promoter as well as the alteration of other genes, such as *TP53*, *PALB2*, *ATM* and *HORMAD1* ^48– 50^. Outside of *BRCA1/2* mutation status, there are no validated predictive biomarkers to identify patients most likely to respond to current therapeutic options in metastatic TNBC (mTNBC). Moreover, this subtype of BC is still suffering from a lack of targetable oncogenic mutations leading to the development of an efficient novel treatment. Platinum-based chemotherapy was associated with a clear benefit among patients harboring germline *BRCA* (g *BRCA*) mutation early after the diagnosis of mBC and showed an improvement in ORR from 33 to 68% when compared with docetaxel ( *P* = 0.03). However, there is no superiority over docetaxel if *BRCA* is not mutated, even in the presence of *BRCA*-ness status (assessed by homologous recombination deficiency assay) ^55, 56^. Besides the cytotoxic chemotherapy, the only approved targeted therapy in mTNBC is the poly (ADP-ribose) polymerase (PARP) inhibitor acting via the concept of synthetic lethality: tumors harboring defects in *BRCA1* and *BRCA2* will fail the reparation of double-strand DNA breaks by homologous recombination and consequently will be highly sensitive to the blockage of single-strand DNA repair mechanisms ^57, 58^. PARP inhibitors also lead to trapping of PARP proteins on DNA in addition to blocking their catalytic action, thus impairing the progression of replication forks ^59, 60^. In this regard, olaparib was compared with chemotherapy (not including platinum salts) in a phase III trial (OlympiAD) enrolling patients with g *BRCA* mutated metastatic TNBC and revealed an improvement in median PFS (mPFS) (7 versus 4.2 months; hazard ratio = 0.58, 95% CI 0.43–0.80; *P* <0.001) and ORR from 28.8 to 59.9% ^61^. Similarly, talazoparib showed a better mPFS compared with non-platinum-based chemotherapy (8.6 versus 5.6 months; hazard ratio = 0.54; 95% CI 0.41–0.71; *P* <0.0001) and an improvement in ORR from 27.7 to 62.2% ^62^. In both trials, platinum-based chemotherapy was not an option in the control arm choices, and the inclusion of patients showing prior progression on a platinum-based chemotherapy was not allowed. In this era of financial toxicity, it would have been interesting to compare platinum (old, cheap, and efficient in g *BRCA* mutated tumors) with PARP inhibitor (new, expensive, and efficient in g *BRCA* mutated tumors). Additionally, PARP inhibitors are known for their synergistic sensitizing effect when given with chemotherapy or ionizing radiation; thus, several trials are evaluating PARP inhibitor combination with other chemo-immunotherapeutic agents: durvalumab plus olaparib ^63^ and pembrolizumab plus niraparib ^64^.

Lurbinectidin (a minor groove DNA binder) was tested in a study by Cruz *et al*. ^65^ in a small series of patients with *BRCA* mutated mBC (n = 54). The ORR was 40.7% and the median duration of response was 6.7 months. Surprisingly, the ORR was 26% in platinum pre-treated patients. These promising results have to be confirmed in a larger trial.

Standard chemotherapy has been revisited with the development of antibody drug conjugates (ADCs). Sacituzumab govitecan (IMMU-132) and glembatumumab vedotin (CDX-011) are two ADCs conjugated with an active metabolite of irinotecan and monomethyl auristatin E (MMAE), respectively ^66, 67^. They showed promising results in early phase II trials with great ORRs and duration of response ^66, 67^ and thus were evaluated in phase III trials: ASCENT (NCT02574455) for IMMU-132 and METRIC (NCT01997333) for CDX-011.

AR was evaluated as a potential target in advanced TNBC ^68^. Its expression varies widely depending on the assay used and cutoff for positivity (immunohistochemistry [IHC] of at least 1% or more than 10%) ^69^. Bicalutamide was the first AR antagonist showing a preliminary activity in heavily pre-treated ER ^−^, AR ^+^ (IHC >10%) BC in a phase II study that enrolled 424 patients with mTNBC. There was a clinical benefit rate (CBR) at 24 weeks of 19%, a PFS of only 12 weeks, and no objective response ^44^. Bonnefoi *et al*. evaluated abiraterone, a 17 alpha-hydroxylase inhibitor, in a phase II trial of 138 patients with diagnosed advanced TNBC; 53 had LAR ^+^ (IHC >10%) ^70^. Abiraterone was associated with a CBR of 20% at 6 months, including one complete response and five patients with stable disease, an ORR of 6.7%, and an mPFS of 2.8 months ^70^. Another AR inhibitor is enzalutamide, which competitively binds to the ligand-binding domain of AR and inhibits AR translocation to the nucleus, recruitment of AR co-factors, and AR binding to DNA. The efficacy of enzalutamide was studied in a single-arm, phase II clinical trial that enrolled patients with advanced AR ^+^ (>0%) TNBC ^71^. One hundred eighteen patients were enrolled, 78 were evaluable, and more than 50% of the patients received enzalutamide as their first or second line. There were promising CBRs of 25% at 16 weeks and 20% at 24 weeks. The CBRs were further improved to 35% at 16 weeks and 29% at 24 weeks in case of AR positivity of more than 10%. Moreover, the mPFS rates were 14.7 weeks in patients with tumors harboring an AR positivity of at least 10% and 12.6 weeks in patients with tumors having AR positivity between 0 and 10% ^71^. A predictive gene expression classifier assay, called Predict-AR, was better able to differentiate responsive patients in the intent-to-treat population: 36% CBR at 24 weeks in Predict-AR ^+^ patients compared with 6% in those whose tumors were Predict-AR ^−^. PFS rates were 16 weeks in patients with Predict-AR ^+^ TNBC and 8 weeks in Predict-AR ^−^ patients. OS was not reached in the genomic test–positive cohort ^72, 73^. Unfortunately, the phase III development of the drug was halted for unknown reasons. Consequently, AR inhibitors are not recommended as standard of care despite showing promising results in a very well-selected population. More definitive trials are needed, and research efforts must determine a better biomarker definition (prognostic, predictive, IHC) ^19^.

**Figure 2.**
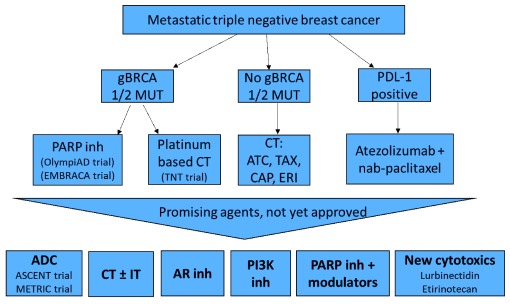
Current standard-of-care treatments in metastatic triple-negative breast cancer and future perspective. ADC, antibody drug conjugate; AR, androgen receptor; ATC, anthracycline; CAP, capecitabine; CT, chemotherapy; ERI, eribublin; g *BRCA* MUT, germline *BRCA* mutation; inh, inhibitor; IT, immunotherapy; PARP, poly (ADP-ribose) polymerase; PDL-1, programmed death ligand 1; PI3K, phosphoinositide-3 kinase; TAX, taxane.

TNBC is characterized by its heterogeneity, aggressive evolution, higher tumor-infiltrating lymphocytes, and potential immunogenicity ^74– 76^. Thus, immunotherapy has been evaluated in many phase I, II, and II trials as a monotherapy or combined with chemotherapy in first-line or a later line of treatment. When the checkpoint inhibitor (CPi) was used earlier in the metastatic setting and prescribed for patients with PDL-1 overexpression, the ORRs were higher ^77– 81^. Likewise, combined immuno-chemotherapy was associated with higher response rates compared with CPi monotherapy ^82– 84^. IMpassion 130 is a phase III trial enrolling 902 patients with metastatic TNBC who had not received prior treatment for metastatic disease. Patients were randomly assigned to one of two groups: standard chemotherapy (nab-paclitaxel) plus atezolizumab (a PDL-1 inhibitor) or nab-paclitaxel plus placebo. With the combination therapy, the risk of disease worsening or death was reduced by 20% in all patients and 38% in the subgroup expressing PDL-1 of at least 1% in immune cells. The mPFS rates were 7.2 months with the combination and 5.5 months with chemotherapy alone in the entire study population (hazard ratio 0.80; *P* = 0.0025). In the PDL-1 ^+^ group, the mPFS rates were 7.5 months with the combination and 5.0 months with chemotherapy alone (hazard ratio 0.62; *P* <0.0001) ^85^. Figure 2 illustrates the current standard of care in TNBC and the future perspective in this subtype of mBC.

## Conclusions

BC is a heterogenic disease that is moving forward as we are further understanding the genomics and driver pathways. mBC survival has drastically improved in luminal and HER2 ^+^ subtypes, but the prognosis of the metastatic triple-negative population remains poor. Precision medicine is promising in many fields of oncology and is slowly arriving to the BC bedside. However, clinicians and researchers are facing many challenges: biomarkers for prediction of resistance and response, the right sequence of available treatments, and how to deal with the financial burden related to these advanced therapeutic options. Liquid biopsy may play a role in the early detection of recurrence, outcome prediction, and understanding of tumor resistance. Many promising discoveries require further validation before becoming a standard of care.
